# The Microbiome in Glioblastoma: Mechanisms, Tumor–Immune Interactions, and Translational Perspectives

**DOI:** 10.1002/mbo3.70431

**Published:** 2026-10-04

**Authors:** Tareq Nayef AlRamadneh, Renuka Jyothi‐S, Priya Priyadarshini‐Nayak, Anima Nanda, Shaker Al‐Hasnaawei, Akanksha Bhatt, Ashish Singh‐Chauhan, Siya Singla, Manoj Kumar Mishra

**Affiliations:** ^1^ Faculty of Allied Medical Sciences, Hourani Center for Applied Scientific Research Al‐Ahliyya Amman University Amman Jordan; ^2^ Department of Biotechnology and Genetics School of Sciences, JAIN (Deemed to be University) Bangalore India; ^3^ Department of Medical Oncology IMS and SUM Hospital, Siksha 'O' Anusandhan Bhubaneswar India; ^4^ Department of Biomedical Sathyabama Institute of Science and Technology Chennai India; ^5^ College of Pharmacy The Islamic University Najaf Iraq; ^6^ Department of Medical Analysis, Medical Laboratory Technique College The Islamic University of Al Diwaniyah Al Diwaniyah Iraq; ^7^ Department of Pharmacy Graphic Era Hill University Dehradun India; ^8^ Adjunct Professor, Center for Promotion of Research Graphic Era Deemed to be University Dehradun India; ^9^ Uttaranchal Institute of Pharmaceutical Sciences, Division of Research and Innovation Uttaranchal University Dehradun India; ^10^ Center for Research Impact and Outcome, Chitkara University Institute of Engineering and Technology Chitkara University Rajpura India; ^11^ Independent Researcher Fitche Ethiopia

**Keywords:** causal inference, glioblastoma, gut microbiota, microbial metabolites, neuroimmune signaling, translational readiness

## Abstract

Despite multimodal treatment, glioblastoma (GBM) remains difficult to control because of diffuse invasion, tumor heterogeneity, immune dysfunction, and frequent recurrence. Increasing attention has focused on whether intestinal microbial communities and their products can modify systemic and central nervous system processes relevant to GBM. This narrative review critically evaluates current evidence linking microbial communities, microbial metabolites, tumor‐associated microbial signals, and host immune responses with GBM biology and clinical translation. PubMed, Scopus, Web of Science, and Google Scholar were searched for English‐language studies published from January 2019 through August 2026, with earlier foundational studies included when necessary. Human cohorts, tumor‐tissue studies, glioma models, microbiota‐transfer experiments, and mechanistic investigations were considered. Preclinical evidence supports several plausible pathways, including changes in short‐chain fatty acid availability, microglial and macrophage states, blood‐brain barrier regulation, systemic immunity, and tryptophan‐related signaling. However, many relevant metabolites may originate from microbial, host, immune, or tumor sources. Human studies remain limited and vulnerable to confounding, reverse causation, treatment effects, and geographic variation. Bacterial nucleic acids and bacteria‐associated HLA‐bound peptides have been detected in brain tumors, but these findings do not establish viable colonization or a gut origin. Low microbial biomass also necessitates rigorous contamination control and orthogonal validation. Microbial influences are biologically plausible modifiers of GBM rather than established drivers. Clinical translation requires longitudinal human studies, source‐resolved analyzes, standardized low‐biomass methods, mechanistic validation, and independent replication. No microbiome‐based biomarker or microbiome‐directed therapy is currently validated for routine GBM care or patient stratification in clinical practice.

## Graphical abstract

Proposed microbiome‐related pathways and translational landscape in glioblastoma. Gut dysbiosis may alter microbial metabolites, inflammatory mediators, barrier integrity, immune signaling, and vagal communication, thereby influencing the GBM neuroimmune microenvironment. Potential downstream effects include changes in microglia/TAMs, astrocytes, T‐cell function, BBB/BTB properties, neuroinflammation, and immune suppression. Human studies, tumor‐associated microbial signals, and animal models provide complementary but still limited evidence, while contamination, uncertain source attribution, poor reproducibility, and lack of validated clinical applications remain major barriers to translation.

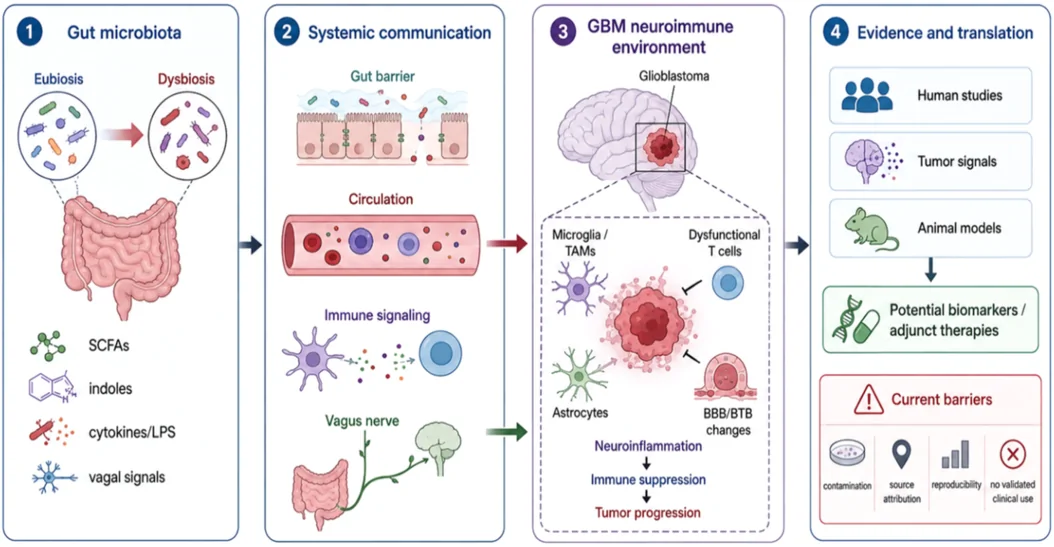

**Abbreviations**
AhR: aryl hydrocarbon receptor
BBB: blood–brain barrier
CNS: central nervous system
FMT: fecal microbiota transplantation
GBM: glioblastoma
GF: germ‐free
HLA: human leukocyte antigen
HPA: hypothalamic–pituitary–adrenal
ICI: immune‐checkpoint inhibitor
NAS: N‐acetylserotonin
SCFAs: short‐chain fatty acids
TAMs: tumor‐associated macrophages
TILs: tumor‐infiltrating lymphocytes
TME: tumor microenvironment
Tregs: regulatory T cells
TrkB: tropomyosin receptor kinase B.

## 1 Introduction

Glioblastoma (GBM) is the most aggressive diffuse primary brain malignancy in adults (Wen et al. 2020). According to the current World Health Organization classification, the term glioblastoma is reserved for diffuse astrocytic tumors that are IDH‐wildtype and classified as CNS WHO grade 4; IDH‐mutant grade 4 tumors are categorized separately as astrocytoma, IDH‐mutant, CNS WHO grade 4 (Louis et al. 2021). The standard treatment for newly diagnosed GBM consists of maximal safe surgical resection followed by radiotherapy with concurrent and adjuvant temozolomide (Weller et al. 2021). The pivotal chemoradiotherapy trial reported median overall survival of approximately 15 months (Stupp et al. 2005), while diffuse infiltration, cellular plasticity, and intratumoral heterogeneity contribute to recurrence and treatment resistance (Neftel et al. 2019). GBM develops within a profoundly immunosuppressive tumor microenvironment dominated by myeloid cells, dysfunctional lymphocytes, inhibitory cytokines, and tumor‐driven immune‐evasion pathways (Andersen et al. 2021a). Randomized phase III trials in recurrent and newly diagnosed GBM have shown that nivolumab‐based immune‐checkpoint inhibition does not improve overall survival in unselected patients (Reardon et al. 2020; Omuro et al. 2023; Lim et al. 2022). These results emphasize that blocking a single checkpoint pathway is insufficient to overcome the layered local and systemic immune dysfunction of GBM and support investigation of additional modulators of antitumor immunity. The gut microbiota has emerged as a systemic regulator of immune homeostasis, inflammation, metabolism, and responses to cancer therapy (Elinav et al. 2019). Communication between the gut and central nervous system occurs through the microbiota‐gut‐brain axis, a bidirectional network integrating neural, endocrine, immune, and metabolic pathways. Gut‐derived signals can reach the CNS through vagal afferents, circulating immune mediators, microbial metabolites, and gut hormones, while autonomic and hypothalamic‐pituitary‐adrenal signaling can modify intestinal motility, barrier function, mucosal immunity, and microbial composition (Cryan et al. 2019; Ramadan et al. 2025a). Microbial products such as short‐chain fatty acids (SCFAs), tryptophan derivatives, and lipopolysaccharide can influence blood‐brain barrier homeostasis, microglial activity, immune‐cell differentiation, and epigenetic regulation (Dalile et al. 2019; Morais et al. 2021). Much of this mechanistic evidence derives from germ‐free animals and non‐neoplastic neurological models, so its direct relevance to human GBM remains incompletely established. Experimental glioma studies provide initial evidence that intestinal microbial perturbation can alter tumor‐associated immunity. Antibiotic‐induced dysbiosis in glioma‐bearing mice has been associated with faster tumor growth, reduced natural‐killer‐cell cytotoxicity, and altered microglial inflammatory programs, while a more recent study linked dietary microbiota‐derived butyrate to glioma control (D'Alessandro et al. 2020; Chen et al. 2025). Human evidence remains substantially less causal. Available studies are predominantly observational and can be confounded by diet, geography, corticosteroids, antibiotics, antiseizure medications, hospitalization, and anticancer treatment (Ishaq et al. 2024; Jiang et al. 2024).

Microbial material has also been detected within human brain tumors. A prospective multi‐institutional study identified bacterial 16S rRNA and lipopolysaccharide signals in tumor, immune, and stromal cells from gliomas and brain metastases, while a separate multi‐omics analysis detected bacterial RNA and lipopolysaccharide in glioma tissue and linked selected signals to immunometabolic and neuronal pathways (Morad et al. 2025; Li and Zhao 2025a). These findings are better described as bacterial or microbial elements than as definitive evidence of a viable, stable intratumoral microbiota. Low‐biomass tissue is particularly vulnerable to reagent and environmental contamination, and sequence detection alone cannot distinguish persistent colonization from transient trafficking or nonviable microbial material (Eisenhofer et al. 2019). Rigorous negative controls, spatial validation, viability assessment, and independent replication are therefore essential. The central issue is therefore not whether microbial signals can influence neural or immune biology in principle, but whether the current evidence is sufficient to establish a reproducible and clinically meaningful microbiota–GBM relationship. This review evaluates that literature through five linked questions: biological plausibility, strength of causal evidence, source attribution, methodological robustness, and translational readiness. It integrates human cohorts, tumor‐tissue studies, glioma‐bearing animal models, and non‐neoplastic mechanistic systems while explicitly distinguishing direct GBM evidence from cross‐disease inference and theoretical models.

## 2 Literature Search Strategy

A structured narrative search was conducted in PubMed/MEDLINE, Scopus, Web of Science Core Collection, and Google Scholar for English‐language literature published from January 2019 through August 2026. Earlier studies were retained when they established foundational mechanisms that remain central to the field, including microglial maturation, blood–brain barrier regulation, short‐chain fatty acid signaling, tryptophan–AhR biology, and standard GBM therapy. Reporting and interpretation were informed by general microbiome best‐practice and STORMS principles (Mirzayi et al. 2021; Knight et al. 2018).

Search combinations included “glioblastoma” or “glioma” with “gut microbiota,” “gut microbiome,” “microbiota–gut–brain axis,” “neuroinflammation,” “microglia,” “macrophage,” “short‐chain fatty acid,” “tryptophan,” “kynurenine,” “aryl hydrocarbon receptor,” “intratumoral bacteria,” “tumor microbiome,” “fungal” or “mycobiome,” “HLA,” “microbial peptide,” “fecal microbiota transplantation,” “probiotic,” “diet,” “artificial sweetener,” and “bacteriophage.” Reference lists of highly relevant reviews and primary studies were also screened to identify additional eligible reports.

Priority was given to peer‐reviewed human studies, prospective tumor‐tissue investigations, glioma‐bearing animal experiments, gnotobiotic or microbiota‐transfer models, mechanistic ex vivo or in vitro studies, and high‐quality reviews used to establish broader biological context. Studies were excluded when they were unrelated to glioma or CNS immune biology, lacked sufficient methodological information for the claim being considered, or duplicated data already represented by a more complete report. Low‐biomass sequencing studies were not automatically excluded; instead, their conclusions were weighted according to contamination control, spatial validation, viability assessment, and independent replication. Because this is a narrative review, study selection was qualitative rather than a PRISMA‐based systematic meta‐analysis.

## 3 Evaluating the Microbiota–GBM Relationship: An Evidence Framework

### 3.1 Levels of Evidence in Microbiota–GBM Research

Microbiota‐GBM research spans evidence types that answer different questions. Cross‐sectional stool studies can identify disease‐associated community patterns but cannot establish temporal direction (Jiang et al. 2022). Tumor‐tissue sequencing can detect microbial nucleic‐acid signals, yet viability and stable colonization require orthogonal validation in low‐biomass tissue (Eisenhofer et al. 2019). Immunopeptidomics and ex vivo T‐cell assays can test antigen presentation and functional recognition, whereas gnotobiotic, transfer, antibiotic‐perturbation, and rescue experiments can test whether manipulation of a microbial exposure changes tumor or immune outcomes (Naghavian et al. 2023; Green et al. 2025). Prospective human studies remain necessary to determine whether microbiota‐GBM relationships are reproducible, temporally informative, and clinically meaningful (Morad et al. 2025).

### 3.2 Association, Biological Plausibility, and Causality

Three levels of inference recur throughout this literature. Biological plausibility is supported when a microbial product has a known host receptor or when a microbial perturbation alters a relevant immune program. Association is supported when a microbial feature covaries with tumor state, treatment response, or survival. Causality requires stronger evidence, including temporality, perturbation, rescue or transfer, source attribution, and preferably replication across models. These distinctions are central to current cancer‐microbiome and GBM‐focused reviews (Sepich‐Poore et al. 2021; Helmink et al. 2019; Nejadrostam et al. 2026).

### 3.3 Confounding, Reverse Causation, and Source Attribution

Source attribution is a second recurring problem. A metabolite detected in plasma or tumor tissue may arise from intestinal microorganisms, host organs, immune cells, or GBM itself; microbial DNA in a low‐biomass tumor may represent viable cells, fragments, transient trafficking, extracellular vesicles, or contamination. Accordingly, this review uses the terms “microbial signal,” “bacterial element,” and “putative tumor‐associated microbial signature” when the data do not establish an active microbial community (Eisenhofer et al. 2019; Fierer et al. 2025; Schorr et al. 2023). The major evidence domains, their interpretive strengths and limitations, and the validation steps required to strengthen microbiota–GBM inference are summarized in Table 1.

**Table 1:** Evidence framework for interpreting microbiota–GBM studies.

Evidence domain	Representative design	What the evidence can establish	Principal limitation	Priority validation step	Reference(s)
Human gut association	Cross‐sectional or longitudinal stool profiling	Association with disease state, treatment, or outcome	Confounding and reverse causation	Prospective multicenter replication with treatment and diet records	(Jiang et al. (2024); Jiang et al. (2022); Nejadrostam et al. (2026))
Tumor‐associated microbial signals	Low‐biomass sequencing plus spatial methods	Presence and localization of microbial material	Contamination and uncertain viability	Negative controls plus orthogonal spatial and viability assays	(Morad et al. (2025); Eisenhofer et al. (2019); Fierer et al. (2025))
Functional antigen evidence	HLA peptide profiling and T‐cell assays	HLA presentation and cross‐reactive recognition	Source and clinical effect unresolved	Matched stool/tumor profiling with functional validation	(Naghavian et al. (2023); Kalaora et al. (2021); Bentzen and Hadrup (2019))
Perturbation models	Antibiotics, defined taxa, diet, or metabolites	Biological response to manipulation	Off‐target and non‐microbial effects	Rescue/recolonization and independent replication	(D'Alessandro et al. (2020); Zhou et al. (2024); Meng et al. (2025))
Transfer models	FMT or humanized‐microbiota mice	Transferability of a community‐associated phenotype	Engraftment and host‐species mismatch	Defined consortia and mechanistic tracing	(Green et al. (2025); Dees et al. (2021); Wang et al. (2025))
Clinical translation	Prospective biomarker or interventional studies	Feasibility, actionability, and patient benefit	No validated GBM‐directed intervention	Standardized trials with safety and target engagement	(Baruch et al. (2021); Duttagupta et al. (2026); Routy et al. (2023))

## 4 The GBM Neuroimmune Environment as a Target of Systemic and Microbial Signals

Microbiota‐associated effects can only be interpreted within the myeloid‐dominant and metabolically constrained GBM immune microenvironment. This section therefore defines the host compartments that systemic microbial signals could plausibly modify rather than treating the microbiota as an established upstream cause of GBM immunosuppression (Andersen et al. 2021a; Klemm et al. 2020; Gao et al. 2025). The GBM tumor microenvironment is a complex cellular ecosystem that supports immune escape, invasion, and therapeutic resistance. The CNS is now understood to exhibit specialized immune regulation rather than complete immune isolation, allowing both resident and peripheral immune cells to participate in brain‐tumor immunity (Smyth and Kipnis 2025; Sampson et al. 2020). Hypoxia, necrosis, nutrient limitation, tumor‐derived cytokines, and altered metabolism further restrict effective antitumor responses. Spatial analyzes and recent mechanistic work underscore that this suppressive environment is heterogeneous and treatment‐responsive rather than immunologically inert (Quail and Joyce 2017; Young et al. 2026).

### 4.1 Myeloid Cell Diversity and Spatial Organization

Tumor‐associated macrophages (TAMs) represent a major immune population in GBM and arise from both brain‐resident microglia and infiltrating monocyte‐derived macrophages. Single‐cell studies show that these populations occupy distinct spatial niches and transcriptional states across tumor regions and disease stages (Klemm et al. 2020; Friebel et al. 2020; Ochocka et al. 2021). The conventional M1/M2 division of GBM‐associated macrophages is therefore overly simplistic. Myeloid cells occupy a continuum of inflammatory, tissue‐remodeling, angiogenic, metabolic, and immunosuppressive states shaped by cellular origin, anatomic niche, tumor genotype, and treatment history (Pombo Antunes et al. 2021; Zheng et al. 2026; Solomou et al. 2024). Glioma cells influence myeloid recruitment and function through mediators including colony‐stimulating factor 1, CCL2, transforming growth factor‐beta, and interleukin‐10. CCL2 can recruit CCR2‐positive monocytic myeloid‐derived suppressor cells and CCR4‐positive regulatory T cells, linking tumor‐derived chemokine signaling to local immune suppression (Chang et al. 2016; Hambardzumyan et al. 2016). TAMs can also release growth factors, proteases, and extracellular‐matrix‐modifying molecules that promote angiogenesis, invasion, and treatment resistance. Human profiling studies further show that glioma‐associated myeloid states differ across tumors and cannot be reduced to a single protumor phenotype (Andersen et al. 2021b; Gabrusiewicz et al. 2016). Because TAM functions are state‐dependent, broad macrophage depletion may remove both tumor‐supporting and potentially protective populations. Current evidence therefore favors selective targeting or reprogramming of defined myeloid pathways over indiscriminate depletion (Zheng et al. 2026; Solomou et al. 2024; Poon et al. 2019).

### 4.2 Regulatory T Cells, T‐Cell Dysfunction, and Systemic Immunosuppression

Regulatory T cells (Tregs) suppress cytotoxic and conventional helper T‐cell responses and can be recruited through CCL2/CCL22‐CCR4 signaling together with other immunosuppressive mediators. Their abundance and prognostic relevance vary across cohorts, so Treg frequency should not be treated as a universal prognostic marker in GBM (Chang et al. 2016; Stepanenko et al. 2024). Recent evidence provides stronger functional support for Treg‐mediated immunosuppression. In a 2025 study, elevated Treg‐associated signatures in human GBM were linked to poorer survival. Selective CD25‐directed Treg depletion in an immunocompetent mouse model promoted CD8‐positive T‐cell expansion and partially controlled tumor growth. The response improved when Treg depletion was combined with PD‐1 blockade. Treg removal also activated intratumoral myeloid cells and increased their phagocytic activity. Similar treatment of patient‐derived GBM fragments depleted Tregs and enhanced CD8‐positive T‐cell activation, although these findings remain preclinical and do not yet demonstrate clinical benefit (Galvez‐Cancino et al. 2025). Effector T cells that reach GBM encounter persistent antigen exposure, inhibitory cytokines, hypoxia, nutrient competition, and checkpoint signaling. GBM‐infiltrating T cells frequently express PD‐1, TIM‐3, LAG‐3, and CTLA‐4 together with transcriptional and functional features of severe exhaustion. Importantly, inhibitory‐receptor expression alone does not define exhaustion; sustained functional impairment and exhaustion‐associated transcriptional or epigenetic programs are also required (Woroniecka et al. 2018). Immune dysfunction also extends beyond the tumor. Treatment‐naive patients and intracranial tumor models have shown sequestration of naive T cells in bone marrow, associated with reduced surface sphingosine‐1‐phosphate receptor 1 and impaired T‐cell egress (Stepanenko et al. 2024; Chongsathidkiet et al. 2018). This combined local and systemic immune dysfunction helps explain why checkpoint inhibition alone has shown limited efficacy in unselected GBM populations (Reardon et al. 2020; Omuro et al. 2023; Lim et al. 2022).

### 4.3 Neurovascular and Metabolic Constraints on Immune Responsiveness

Hypoxia, nutrient competition, altered intermediary metabolism, and cytokine gradients shape immune‐cell states in GBM. Single‐cell and spatial studies demonstrate niche‐dependent myeloid programs, while recent work links microglial metabolism and IL‐6 signaling to immune suppression and therapeutic resistance (Young et al. 2026; Ochocka et al. 2021; Billingham et al. 2026). This context is important when microbial metabolites are proposed to alter immune polarization because the same signal may have different consequences at the invasive margin, perivascular niche, or hypoxic core.

## 5 The Gut–Brain Axis and Microbial Modulation of Neuroinflammation

### 5.1 Barrier and Systemic Inflammatory Signaling

The microbiota‐gut‐brain axis is a bidirectional communication network linking the gastrointestinal tract with the CNS through immune, metabolic, endocrine, neural, and barrier‐associated signals. Microbial metabolites, structural products, gut hormones, the enteric nervous system, and autonomic pathways participate in gut‐to‐brain communication, while CNS‐derived autonomic and hypothalamic‐pituitary‐adrenal signals can modify intestinal physiology and microbial composition (Cryan et al. 2019; Morais et al. 2021). Evidence from non‐neoplastic neurological and neurodevelopmental conditions indicates that intestinal dysbiosis can accompany barrier dysfunction, systemic inflammation, and altered neuroactive or metabolic signaling. Microbiome‐directed interventions have shown preliminary effects in selected non‐GBM settings, but these findings are disease‐specific and cannot be directly extrapolated to glioblastoma (Ramadan et al. 2025a; Hetta et al. 2025a; Keane et al. 2025). Dysbiosis may increase systemic exposure to microbial products such as lipopolysaccharide, which can activate pattern‐recognition receptors and peripheral cytokine signaling. These systemic signals may influence endothelial and glial states, but direct entry of gut‐derived LPS into GBM tissue and a causal effect on human tumor growth have not been demonstrated (Cryan et al. 2019; Logsdon et al. 2018).

### 5.2 SCFA‐Dependent Glial and Myeloid Regulation

SCFAs, mainly acetate, propionate, and butyrate, are produced by microbial fermentation and can influence immune differentiation, metabolism, epigenetic regulation, and endothelial function (Dalile et al. 2019). Experimental studies do not support describing SCFAs as agents that “breach” the blood‐brain barrier; germ‐free mice instead show increased permeability, while microbial colonization or butyrate exposure can restore selected tight‐junction features (Logsdon et al. 2018; Braniste et al. 2014). The microbiota also contributes to microglial maturation. Germ‐free mice exhibit immature microglial transcriptional and functional phenotypes, and colonization can reverse selected abnormalities. These foundational findings establish a microbiota‐microglia relationship in non‐tumor models rather than a direct GBM mechanism (Erny et al. 2015; Thion et al. 2018).

More direct evidence has emerged from an orthotopic GBM model. Antibiotic‐induced dysbiosis was associated with reduced SCFA concentrations in blood and tumor tissue, increased tumor progression, and a shift toward macrophage markers classified by the investigators as M2‐like. Oral SCFA supplementation increased glycolytic activity in tumor‐associated macrophages, enhanced markers categorized as M1‐like, and improved tumor‐related outcomes. This study provides GBM‐specific preclinical evidence for a microbiota–SCFA–myeloid‐cell pathway. However, it represents a single animal study, and its binary M1/M2 classification does not capture the broader diversity of macrophage states found in human GBM (Zhou et al. 2024). Broader syntheses of microbiota‐microglia and neuroimmune communication reinforce this mechanistic plausibility while emphasizing strong dependence on developmental stage, host context, and disease model (Abdel‐Haq et al. 2019; Fung et al. 2017).

### 5.3 Tryptophan–AhR Signaling

Gut microorganisms generate tryptophan‐derived indoles that can activate the aryl hydrocarbon receptor (AhR). In experimental autoimmune encephalomyelitis, microbiota‐derived tryptophan metabolites modulated astrocyte activity and limited CNS inflammation, demonstrating a glial regulatory mechanism that remains context‐dependent and cannot be assumed to operate identically in GBM (Rothhammer et al. 2016). The kynurenine pathway also intersects with AhR signaling. However, kynurenine in the GBM microenvironment may be generated by tumor cells, immune cells, or systemic host metabolism and is not necessarily microbial in origin. The relative contribution of gut‐derived indoles and host‐derived kynurenine to AhR activity in GBM remains unresolved (Guastella et al. 2018). Detailed discussion of the metabolic and epigenetic effects of these compounds is provided in the following metabolite‐focused section to avoid repetition. Microbial indoles, host kynurenine metabolism, and tumor‐intrinsic TDO/IDO activity converge on AhR biology but arise from biologically distinct sources. Direct GBM evidence supports tumor‐associated kynurenine‐AhR signaling, whereas a quantitatively important contribution from gut‐derived AhR ligands to the human GBM microenvironment remains unproven (Takenaka et al. 2019; Zelante et al. 2013; Opitz et al. 2011).

### 5.4 Neural and Neuroendocrine Routes

The vagus nerve provides a neural route through which intestinal chemical and mechanical signals can influence brain function. Microbial metabolites and gut hormones may be detected directly or indirectly by vagal afferents. The CNS can reciprocally alter intestinal physiology through parasympathetic, sympathetic, and HPA‐axis signaling (Cryan et al. 2019). Although vagal and hypothalamic‐pituitary‐adrenal pathways are established components of microbiota‐gut‐brain communication, direct evidence that these routes regulate GBM growth, tumor‐associated edema, seizure activity, or immune‐cell recruitment is lacking. Their contribution to glioblastoma should therefore be tested through experimental manipulation coupled to tumor, immune, and microbiome measurements. Vagal, autonomic, and neuroendocrine signaling are well‐established components of general microbiota‐gut‐brain communication, but their direct contribution to GBM biology remains a testable hypothesis rather than an established mechanism (Ramadan et al. 2025a; Fung et al. 2017; Bonaz et al. 2018).

### 5.5 Human Evidence and Its Boundaries

Human data remain limited. In a pilot cohort of 27 patients with malignant glioma, 32 with meningioma, and 41 healthy controls, brain‐tumor patients showed lower microbial diversity than healthy participants; *Fusobacterium* and *Akkermansia* were enriched in the glioma group, whereas several SCFA‐associated taxa were reduced. The exploratory microbial panel was derived within the same dataset and was not externally validated (Jiang et al. 2022). Current evidence therefore supports the microbiota as a potential modifier, rather than an established driver, of GBM‐associated neuroinflammation. Longitudinal studies should combine microbial sequencing with metabolomic measurements, immune profiling, medication and dietary records, and molecular classification of the tumor. The principal pathways and their current levels of evidence are summarized in Figure 1. Recent GBM‐focused reviews similarly conclude that human microbiome evidence is expanding but remains heterogeneous, treatment‐sensitive, and substantially less causal than the animal literature (Ishaq et al. 2024; Jiang et al. 2024; Green et al. 2024).

**Figure 1:**
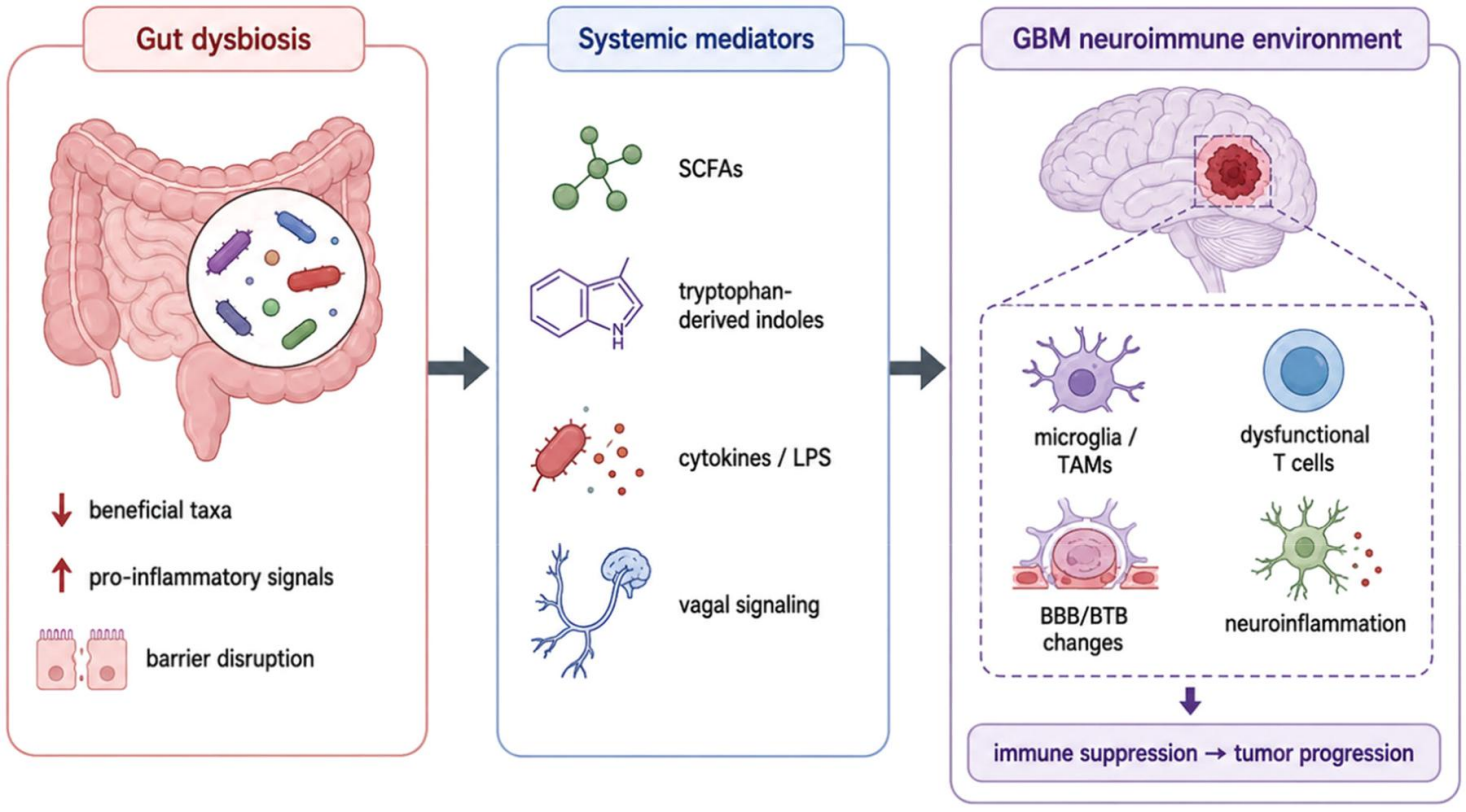
Proposed gut microbiota–mediated pathways influencing the glioblastoma neuroimmune microenvironment. Gut dysbiosis may reduce beneficial taxa, increase pro‐inflammatory signals, and impair intestinal barrier integrity. Resulting systemic mediators, including short‐chain fatty acids (SCFAs), tryptophan‐derived indoles, cytokines, lipopolysaccharide (LPS), and vagal signals, may influence microglia and tumor‐associated macrophages (TAMs), T‐cell function, blood‐brain/blood‐tumor barrier (BBB/BTB) properties, and neuroinflammatory signaling. These changes may contribute to immune suppression and GBM progression.

## 6 Microbial, Host, and Tumor‐Derived Metabolites in GBM

A central distinction for this review is that a metabolite relevant to GBM is not necessarily microbiota‐derived. Source attribution is therefore treated as part of the mechanism rather than an afterthought.

### 6.1 SCFAs and Metabolic–Epigenetic Regulation

Microbial metabolites provide an interface between the intestinal microbiota and host physiology, influencing immune‐cell differentiation, cellular metabolism, transcriptional regulation, and chromatin organization (Dalile et al. 2019; Ramos‐Molina et al. 2019; Rooks and Garrett 2016). In GBM, SCFAs, kynurenine/AhR signaling, and polyamines intersect with experimentally relevant tumor and immune pathways. Importantly, these metabolites may arise from microbial, host, immune‐cell, or tumor‐intrinsic sources, making source attribution part of the mechanism rather than an afterthought (Zhou et al. 2024; Takenaka et al. 2019; Kay et al. 2025).

Anaerobic intestinal bacteria, including *Faecalibacterium prausnitzii* and *Roseburia* species, generate acetate, propionate, and butyrate from dietary carbohydrates. SCFAs act through G‐protein‐coupled receptors, metabolic substrate availability, and epigenetic mechanisms; butyrate and, to a lesser extent, propionate can inhibit histone deacetylases, whereas acetate primarily contributes to acetyl‐CoA metabolism (Koh et al. 2016; Luu et al. 2021). In human glioma cell lines, sodium butyrate has increased histone acetylation, reduced proliferation, and promoted apoptosis or senescence, including in combination experiments. These data establish direct butyrate‐responsive cellular pathways in vitro, but the millimolar exposures used do not demonstrate that microbiota‐derived butyrate reaches human GBM tissue at comparable concentrations (Nakagawa et al. 2018; Qin et al. 2020; Majchrzak‐Celińska et al. 2021).

More recent preclinical evidence suggests that SCFA availability may influence the GBM immune microenvironment. In an orthotopic mouse model, antibiotic‐associated gut dysbiosis reduced SCFA concentrations in blood and tumor tissue and was accompanied by accelerated tumor progression. Oral SCFA supplementation altered the metabolic and phenotypic state of tumor‐associated macrophages and improved tumor‐related outcomes. This study supports a microbiota–SCFA–GBM connection in mice, but it remains a single preclinical investigation. Its use of binary M1/M2 macrophage terminology also does not fully represent the diverse myeloid states observed in human GBM. Detailed immune effects of this study were discussed in the preceding section and are not repeated here (Zhou et al. 2024). SCFA effects depend on concentration, target cell, metabolic state, and route of exposure. Butyrate can inhibit proliferation in some tumor‐cell systems while supporting regulatory or anti‐inflammatory immune programs in other contexts; SCFAs should therefore not be described as uniformly antitumor molecules (Dalile et al. 2019; Koh et al. 2016; Luu et al. 2021). Foundational non‐tumor studies link SCFAs to host epigenetic programming, regulatory T‐cell differentiation, and hematopoietic responses (Krautkramer et al. 2016; Arpaia et al. 2013; Trompette et al. 2014). These mechanisms strengthen biological plausibility, but physiologic SCFA concentrations and target engagement within human GBM remain insufficiently characterized. Complementary mechanistic studies show that SCFAs can directly regulate immune‐cell function and that microbiota‐derived butyrate contributes to regulatory T‐cell differentiation and colonic Treg homeostasis (Corrêa‐Oliveira et al. 2016; Furusawa et al. 2013; Smith et al. 2013). These immune effects provide mechanistic context rather than GBM‐specific evidence.

### 6.2 Tryptophan, Kynurenine, and AhR

Dietary tryptophan can enter microbial and host metabolic pathways. Gut microorganisms produce indole derivatives, whereas host and tumor cells generate kynurenine through IDO1 and TDO2; both classes of metabolites can engage AhR‐dependent biology (Rothhammer et al. 2016; Roager and Licht 2018; Platten et al. 2019). Microbiota‐derived indoles regulate astrocytic and immune‐cell activity in non‐neoplastic CNS models. In contrast, direct GBM evidence more strongly supports kynurenine produced within the tumor microenvironment as an AhR ligand. In experimental GBM, tumor‐cell‐derived kynurenine activated AhR in tumor‐associated macrophages, promoted CCR2‐dependent recruitment and CD39‐associated immunosuppression, and reduced effective CD8‐positive T‐cell activity. Genetic interference with tumor kynurenine production delayed tumor growth in mice, supporting a tumor‐intrinsic kynurenine‐AhR pathway rather than a demonstrated gut source (Takenaka et al. 2019). Additional glioma studies indicate that TDO2‐dependent kynurenine production can support AhR‐related survival and immunosuppressive signaling. The direction of AhR biology therefore depends on ligand source and cellular context: microbial indoles can be anti‐inflammatory in some CNS settings, whereas tumor‐derived kynurenine can support glioma survival and immune suppression (Zelante et al. 2013; Zhong et al. 2022; Lamas et al. 2016).

### 6.3 Polyamine Metabolism

Polyamines, including putrescine, spermidine, and spermine, participate in nucleic‐acid stabilization, translation, proliferation, redox regulation, and chromatin organization. Gut microorganisms can contribute to intestinal and circulating polyamine pools, but GBM cells and host tissues also synthesize these metabolites through ornithine decarboxylase‐dependent pathways (Ramos‐Molina et al. 2019; Wei et al. 2025). A recent GBM study showed that tumor‐cell‐derived spermidine was elevated in the tumor microenvironment and promoted tumor aggressiveness through immune‐dependent suppression of CD8‐positive T‐cell abundance and cytotoxicity. ODC knockdown prolonged survival, and lower spermidine concentrations were associated with more favorable patient outcomes (Kay et al. 2025). ODC‐dependent polyamine synthesis also supports the self‐renewal and tumorigenic properties of glioblastoma stem‐like cells; genetic or pharmacologic disruption of this pathway reduces stem‐associated programs in experimental models (Wei et al. 2025). Its relationship to gut microbial polyamine production has not been directly established. Polyamine biology also intersects with the myeloid compartment: experimental GBM work has shown that polyamine‐dependent pH buffering can support myeloid survival and immunosuppression (Miska et al. 2021). This strengthens the relevance of the pathway while again underscoring that tumor and host synthesis must be separated from microbial contribution.

### 6.4 The Source‐Attribution Problem

Metabolite signaling in GBM arises from overlapping microbial, systemic, immune, and tumor‐intrinsic pathways. A metabolite detected in plasma, cerebrospinal fluid, or tumor tissue cannot be assigned to the gut microbiota solely because intestinal microorganisms are capable of producing it; this is particularly important for acetate, kynurenine, and polyamines (Zhou et al. 2024; Takenaka et al. 2019; Kay et al. 2025). Future studies should combine stool metagenomics with targeted metabolomics in plasma, cerebrospinal fluid, tumor tissue, and matched peritumoral tissue. Stable‐isotope tracing could help distinguish dietary and microbial metabolites from those synthesized by GBM or immune cells. Measurements should also account for diet, antibiotics, corticosteroids, temozolomide, antiseizure medications, and renal or hepatic metabolism. SCFA‐, kynurenine‐AhR‐, and polyamine‐dependent pathways are biologically relevant in experimental GBM, but microbiota‐derived metabolites are not validated biomarkers or therapeutic targets in patients. Clinical translation requires physiologically relevant dosing, source confirmation, longitudinal sampling, and demonstration of target engagement within the human GBM microenvironment (Zhou et al. 2024; Zhong et al. 2022; Wei et al. 2025).

## 7 Experimental Tests of Microbiota–GBM Causality

Experimental intervention provides the clearest route from association toward causality, but different manipulations answer different questions. Depletion tests dependence on a broad microbial ecosystem, transfer tests whether a community‐associated phenotype is transmissible, and defined‐taxon or metabolite rescue can narrow the responsible mechanism.

### 7.1 Antibiotic Perturbation and Metabolite Rescue

Manipulation of the intestinal microbiota provides more direct evidence that microbial communities can influence glioma‐associated immunity. One of the earliest glioma‐specific studies used chronic antibiotic exposure in mice bearing intracranial GL261 tumors. Antibiotic treatment altered intestinal bacterial composition, reduced cytotoxic natural killer‐cell subsets, modified inflammatory and homeostatic proteins in microglia, and was accompanied by increased intracranial tumor growth (D'Alessandro et al. 2020). A subsequent orthotopic model similarly showed accelerated tumor progression following antibiotic‐induced dysbiosis. Reduced short‐chain fatty acid concentrations were detected in blood and tumor tissue, while oral SCFA supplementation partly reversed tumor‐associated changes, increased macrophage glycolytic activity, and improved tumor‐related outcomes (Zhou et al. 2024). These findings support a microbiota–metabolite–myeloid‐cell pathway but do not exclude direct pharmacological or metabolic effects of antibiotics and SCFAs.

### 7.2 Gnotobiotic, Humanized‐Microbiome, and FMT Models

Fecal microbiota transplantation and gnotobiotic approaches provide a higher level of causal inference than cross‐sectional profiling. Distinct human‐derived microbial communities have produced different immune‐cell states and differential responses to anti‐PD‐1 treatment in murine glioma models (Green et al. 2025; Dees et al. 2021). More direct transfer experiments compared microbiota from patients with high‐grade glioma with microbiota from healthy participants. Mice receiving high‐grade glioma‐associated microbiota developed faster‐growing tumors. *Eisenbergiella*, *Mailhella*, and *Merdimonas* were enriched in these recipient animals, and *Merdimonas* abundance correlated with sphingosine‐related metabolites (Wang et al. 2025). Transfer experiments support the transmissibility of community‐associated phenotypes but do not establish that a single bacterial taxon or metabolite is sufficient to drive glioma progression. Donor selection, recipient genotype, engraftment, diet, and co‐transfer of non‐bacterial components can materially influence FMT and humanized‐microbiome models (Moreno‐Indias et al. 2020; Lam et al. 2022).

### 7.3 Defined Taxa, Probiotics, and Immunotherapy

Defined microbial interventions provide a more controlled approach than whole‐community transfer. In an orthotopic GL261 model, combined administration of *Bifidobacterium lactis* and *Lactobacillus plantarum* reduced tumor volume, prolonged survival, improved intestinal‐barrier integrity, and altered tumor PI3K/Akt‐associated signaling (Wang et al. 2022). However, the relative contributions of direct probiotic effects, community restructuring, microbial metabolites, intestinal‐barrier restoration, and systemic immunity remain difficult to distinguish.

Microbial metabolites may also influence immunotherapy responses. Sodium butyrate inhibited glioma‐cell growth in vitro and increased PD‐L1 expression through PI3K/Akt‐associated signaling. In an orthotopic model, sodium butyrate combined with anti‐PD‐1 treatment reduced tumor burden and prolonged survival more effectively than either treatment alone (Li et al. 2025b). Because sodium butyrate also functions directly as a histone deacetylase inhibitor, these effects cannot be attributed exclusively to alteration of the microbiome. The broader oncology literature shows that gut microbial composition can correlate with or modify checkpoint‐inhibitor activity in melanoma and epithelial cancers. These findings provide proof of principle but are not interchangeable with GBM, where lymphocyte scarcity and checkpoint resistance are more profound (Gopalakrishnan et al. 2018; Routy et al. 2018; Matson et al. 2018).

### 7.4 Dietary Exposures and Artificial Sweeteners

Dietary experiments further illustrate the context dependence of microbiome‐mediated effects. A short‐term high‐glucose drink prolonged survival in GL261‐bearing mice, altered gut microbial composition, enriched *Desulfovibrio*naceae, and enhanced T‐cell‐mediated antitumor immunity. *Desulfovibrio vulgaris* contributed to the observed interaction with anti‐PD‐1 therapy, including an increase in cytotoxic CD4‐positive T cells (Kim et al. 2023). This highly specific experimental regimen should not be interpreted as evidence that high dietary sugar intake is therapeutically beneficial in patients with GBM. Non‐nutritive sweeteners provide another example of context‐dependent effects. Their influence on the microbiome differs according to compound, dose, duration, host metabolism, background diet, and baseline microbial composition (Hetta et al. 2025b). In a 2025 GBM mouse study, aspartame altered fecal microbial composition and was associated with extensive changes in tumor mRNA expression and N6‐methyladenosine modification, including increased expression of MYC, CDKN1A, and TGFB1. However, it did not significantly increase tumor growth (Meng et al. 2025).

### 7.5 What Preclinical Models Can and Cannot Prove

Most microbiome–glioma experiments rely on GL261 or other implanted murine tumor models. Although these models permit immune analysis in immunocompetent hosts, they do not fully reproduce the molecular heterogeneity, diffuse invasion, treatment history, and profound immune suppression characteristic of human IDH‐wildtype GBM (Slika et al. 2023). Microbiome models introduce additional limitations. Germ‐free mice display developmental abnormalities in both immune and nervous systems, whereas antibiotic‐treated animals retain a reduced and compositionally altered microbiota rather than becoming truly germ‐free. Humanized microbiome models are also imperfect because human microorganisms may not engraft stably and may behave differently under murine diet, anatomy, bile‐acid metabolism, and immune pressure (Moreno‐Indias et al. 2020). FMT can additionally transfer viruses, fungi, metabolites, dietary molecules, and host‐derived components together with bacteria (Lam et al. 2022).

Experimental design should therefore account for sex, age, animal supplier, cage effects, diet, circadian timing, antibiotic pharmacology, and baseline microbial composition (Gozalo and Elkins 2023). Studies should report microbial engraftment, absolute microbial load, metabolite concentrations, tumor burden, immune‐cell phenotypes, and survival rather than relying only on relative taxonomic abundance. Stronger causal inference will require combinations of depletion, transfer, recolonization, defined microbial consortia, and metabolite rescue. Longitudinal sampling of stool, blood, cerebrospinal fluid, and tumor tissue, together with stable‐isotope tracing, metabolomics, single‐cell profiling, and spatial analysis, could determine whether specific microbial functions reach and modify the GBM microenvironment. No current preclinical evidence justifies recommending FMT, probiotics, high‐glucose intake, artificial sweeteners, SCFA supplementation, or other microbiome‐directed interventions as treatment for patients with GBM. The strongest findings remain model‐dependent and require independent replication, physiologically relevant exposure assessment, and prospective human validation (Zhou et al. 2024; Wang et al. 2025; Li et al. 2025b). The main experimental approaches used to interrogate microbiota–GBM interactions, together with the level of causal inference that each can support, are outlined in Figure 2.

**Figure 2:**
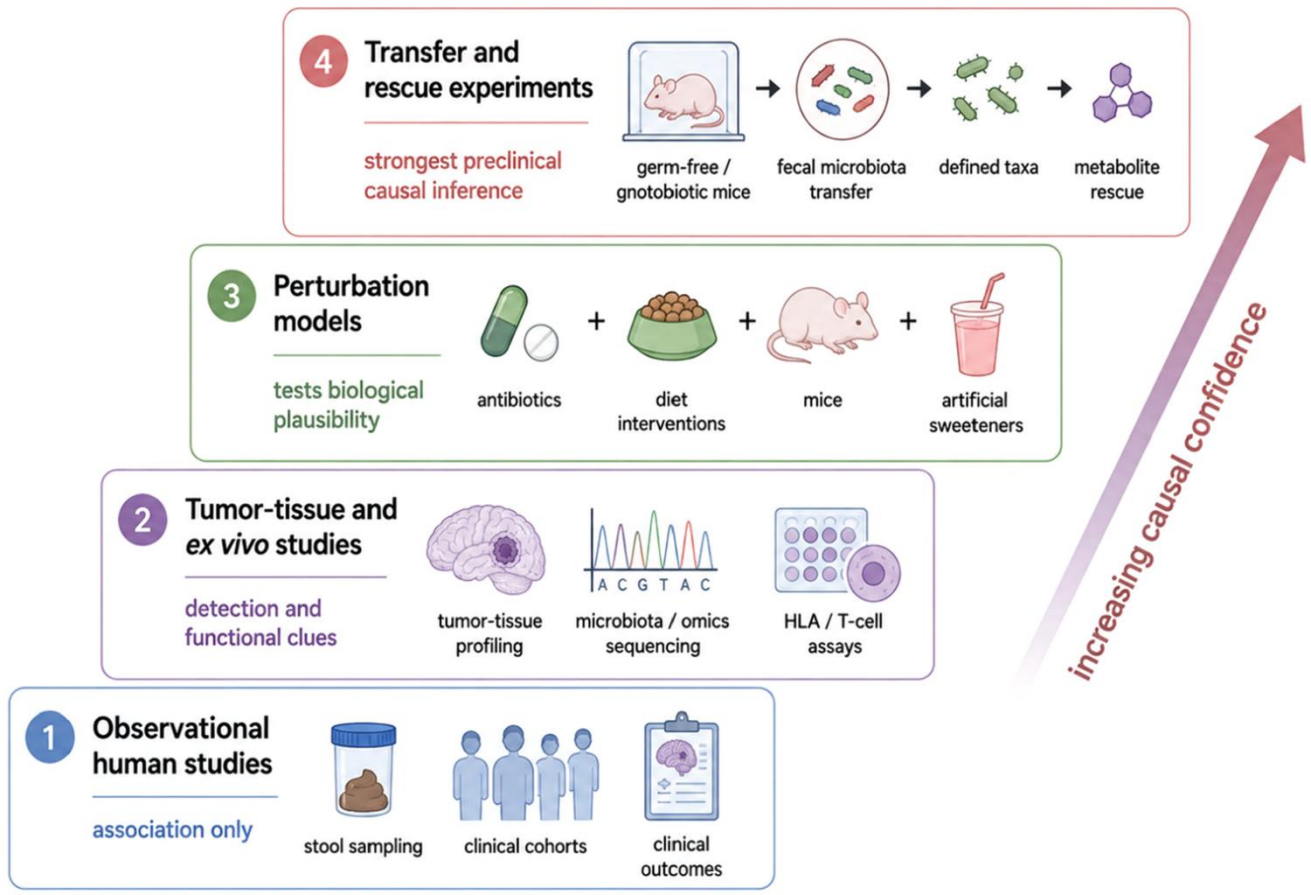
Hierarchy of experimental approaches for evaluating microbiota–GBM causality. Evidence progresses from observational human studies showing associations, through tumor‐tissue and ex vivo analyzes providing molecular and functional clues, to perturbation models testing biological plausibility. Germ‐free or gnotobiotic models, fecal microbiota transfer, defined taxa, and metabolite‐rescue experiments provide the strongest preclinical support for causal inference, although confirmation in human GBM remains necessary.

## 8 Tumor‐Associated Microbial Signals: Presence, Function, or Contamination?

### 8.1 Bacterial Signals: Detection Versus Colonization

The healthy brain has traditionally been considered free of resident microbial communities. Recent studies have nevertheless detected low‐abundance microbial material in several tumor types, including primary and metastatic brain tumors. These observations support the presence of bacterial elements in some tumors but do not establish a stable, biologically active GBM microbiota (Morad et al. 2025; Nejman et al. 2020). Sequencing‐based tumor microbiome studies remain particularly vulnerable to low‐biomass artifacts, including reagent contamination, environmental DNA, index misassignment, and computational misclassification. Retrospective taxonomic profiles should therefore be treated as exploratory unless supported by appropriate controls and orthogonal validation (Eisenhofer et al. 2019; Gihawi et al. 2023; Ge et al. 2025). Stronger brain‐tumor‐specific evidence comes from a prospective multi‐institutional study of 243 samples from 221 patients, including 113 gliomas and 55 brain metastases. The investigators combined RNA‐based fluorescence in situ hybridization, immunohistochemistry, spatial imaging, 16S rRNA sequencing, metagenomics, and culturomics. Bacterial 16S rRNA and lipopolysaccharide signals were detected in subsets of glioma and metastatic samples, including tumor, immune, and stromal cells. Sequencing identified signals assigned to genera including *Fusobacterium*, *Prevotella*, *Veillonella*, *Capnocytophaga*, and *Enterococcus*, with partial overlap between tumor‐associated signals and matched oral or gut communities. Standard culture did not recover a readily cultivable bacterial community, the signals were sparse and heterogeneous, and no independent association with clinical outcome was established (Morad et al. 2025).

### 8.2 Fungal Signals and the Question of a GBM Mycobiome

Evidence for fungi within GBM is weaker than the evidence for bacterial elements. Pan‐cancer analyzes have identified low‐abundance fungal signals across multiple malignancies, but the most robust patterns have been reported in gastrointestinal, pancreatic, and lung cancers rather than in molecularly defined GBM (Narunsky‐Haziza et al. 2022).

In the pan‐cancer analysis by Dohlman et al. lower‐grade glioma samples largely served as a low‐fungal‐biomass comparison group. *Candida albicans* and *Saccharomyces cerevisiae* reads were largely absent from brain samples, while widely distributed *Malassezia globosa* reads showed a pattern compatible with biological or technical contamination; the strongest validated findings concerned gastrointestinal and lung cancers (Dohlman et al. 2022). At present, no reproducible evidence demonstrates viable fungal colonization, a stable GBM‐associated mycobiome, or a causal fungal contribution to glioblastoma progression. Confirmation would require fungal‐specific spatial detection, evidence of transcriptional activity or viability, and replication in independent GBM cohorts (Fierer et al. 2025).

### 8.3 HLA‐Presented Bacteria‐Associated Peptides and Cross‐Reactivity

Molecular mimicry occurs when microbial and host‐derived peptides share structural or sequence features that permit recognition by the same T‐cell receptor. In cancer, such cross‐reactivity may connect previous microbial exposure with immune recognition of tumor‐associated or tumor‐specific antigens (Tagliamonte and Buonaguro 2022). Bacteria‐derived peptides have been identified on HLA class I and II molecules in melanoma, demonstrating that microbial proteins can enter tumor‐associated antigen‐processing pathways (Kalaora et al. 2021). In GBM, the most detailed evidence comes from an immunopeptidomic analysis of primary and recurrent tumors from 19 patients. Between 5 and 54 unique bacterial sequences bound to HLA class II were identified per tumor, yielding 344 distinct bacteria‐associated peptides across the cohort. These sequences were assigned predominantly to proteins from Proteobacteria and Firmicutes, and small numbers of HLA class II‐bound bacterial peptides were also detected in patient‐derived GBM cell lines (Naghavian et al. 2023). These findings show that bacteria‐associated peptide sequences can enter the HLA immunopeptidome of GBM tissue and cultured tumor cells, but they do not establish the anatomical source of the microbial material or viable intratumoral colonization. Tumor cells, local antigen‐presenting cells, or both may contribute to presentation. In the same study, bacteria‐associated peptides were recognized by GBM‐infiltrating T cells, although directly eluted bacterial peptides stimulated bulk tumor‐infiltrating lymphocytes only weakly. More detailed functional experiments centered largely on a patient‐derived CD4‐positive T‐cell clone that recognized both a mutated GBM antigen and several microbial peptides, providing proof of HLA‐restricted cross‐reactivity without establishing a general clinical effect.

These experiments support HLA‐restricted microbial‐tumor T‐cell cross‐reactivity but do not demonstrate that commensal microorganisms routinely prime effective antitumor immunity in GBM. Sequence similarity alone is insufficient for molecular mimicry; natural processing, compatible HLA presentation, and recognition by the same T‐cell receptor are required (Bentzen and Hadrup 2019; Tagliamonte and Buonaguro 2022). Microbial–tumor cross‐reactivity could theoretically influence antitumor immunity in several directions. Pre‐existing memory T cells generated through exposure to commensal or pathogenic microorganisms might recognize structurally related tumor antigens and provide accelerated immune responses. Proof of principle has been demonstrated outside GBM: a peptide encoded by *Bifidobacterium breve* induced CD8‐positive T cells that cross‐recognized a related tumor antigen and reduced melanoma growth in mice (Bessell et al. 2020). However, cross‐reactivity is not necessarily beneficial. Repeated exposure to related microbial antigens may generate low‐avidity, dysfunctional, or regulatory responses, and cross‐reactive T‐cell receptors may also recognize peptides expressed in healthy tissues (Bentzen and Hadrup 2019). Current GBM evidence does not establish whether bacteria‐associated peptide recognition improves tumor control, contributes to immune dysfunction, or has no measurable influence on clinical outcome. The anatomical origin of these microbial antigens is also unresolved. The bacterial species assigned to identified peptides included both pathogenic and commensal organisms, but matched intestinal microbiota were not analyzed. HLA‐presented bacterial peptides therefore cannot currently be assumed to originate from the gut microbiota. Demonstrating such a relationship would require matched stool, blood, and tumor sampling, strain‐ or gene‐level microbial identification, HLA immunopeptidomics, and functional T‐cell receptor analysis (Naghavian et al. 2023). HLA‐presented microbial peptides may eventually expand the repertoire of antigens considered for personalized immunotherapy, including peptide or mRNA vaccines and engineered cross‐reactive T cells. These applications remain experimental. Candidate receptors would require extensive off‐target screening against the human proteome and primary healthy tissues because cross‐reactivity with essential self‐antigens could produce serious toxicity (Bentzen and Hadrup 2019).

### 8.4 Possible Routes of Entry and Biological Interpretation

Several mechanisms could account for microbial material in brain tumors, including transient hematogenous entry of microbial fragments or extracellular vesicles and transport within phagocytic immune cells. These routes remain hypothetical in GBM and should not be interpreted as established microbial trafficking pathways (Morad et al. 2025; Schorr et al. 2023).

Necrosis and impaired local immunity may provide a permissive environment, but the failure to recover a readily cultivable bacterial community from prospective brain‐tumor samples argues against assuming conventional bacterial colonization. Spatial analyzes have associated bacterial 16S‐positive regions with antimicrobial and immunometabolic transcriptional programs. These relationships are correlative and do not establish whether microbial signals caused the local transcriptional state or accumulated within regions that were already biologically altered (Morad et al. 2025). Cross‐cancer studies show that defined bacterial species can influence inflammation, genotoxicity, immune remodeling, or therapy response in gastrointestinal and pancreatic malignancies. Comparable bacterial drivers have not been established in GBM, so extracranial mechanisms should be used to generate hypotheses rather than to infer bacteria‐driven gliomagenesis (Ramadan et al. 2026; Kostic et al. 2013; Pushalkar et al. 2018).

### 8.5 Methodological Standards for Low‐Biomass Brain‐Tumor Studies

Brain tumors contain very low microbial biomass, so small amounts of external DNA can materially distort taxonomic profiles. Extraction blanks, no‐template controls, environmental controls, randomized processing, batch documentation, and appropriate decontamination procedures should therefore accompany tumor samples from collection through bioinformatic analysis (Eisenhofer et al. 2019; Fierer et al. 2025). Reagent‐derived bacterial DNA can dominate low‐biomass sequencing datasets, and validated statistical decontamination methods can help identify contaminant sequences when appropriate controls and batch information are available (Salter et al. 2014; Glassing et al. 2016; Davis et al. 2018). Computational filtering should complement, not replace, experimental negative controls.

Sequence detection should be paired with orthogonal methods such as RNA‐based in situ hybridization, organism‐specific probes, microbial‐component staining, digital PCR, high‐resolution imaging, culture, and viability‐sensitive assays. Absolute microbial load and matched blood, oral, stool, tumor, and adjacent‐tissue sampling can further strengthen source attribution (Morad et al. 2025; Schorr et al. 2023). The terms “microbiota” and “microbiome” should be reserved for settings in which a diverse and biologically active community is supported by adequate evidence. Until viability, replication, and function are demonstrated, “microbial signals,” “bacterial elements,” or “putative tumor‐associated microbial signatures” are more accurate descriptions for most GBM findings (Fierer et al. 2025). The 2024 retraction of the TCGA‐based cancer microbial‐signature study illustrates why low‐biomass computational findings require independent validation. The retracted report is not used as supporting evidence here; interpretation instead relies on dedicated decontamination studies, reanalysis of TCGA microbial content, and the formal retraction record (Gihawi et al. 2023; Ge et al. 2025; Poore et al. 2024). A conceptual framework for interpreting tumor‐associated microbial findings in GBM, from low‐biomass detection to functional and translational inference, is presented in Figure 3.

**Figure 3:**
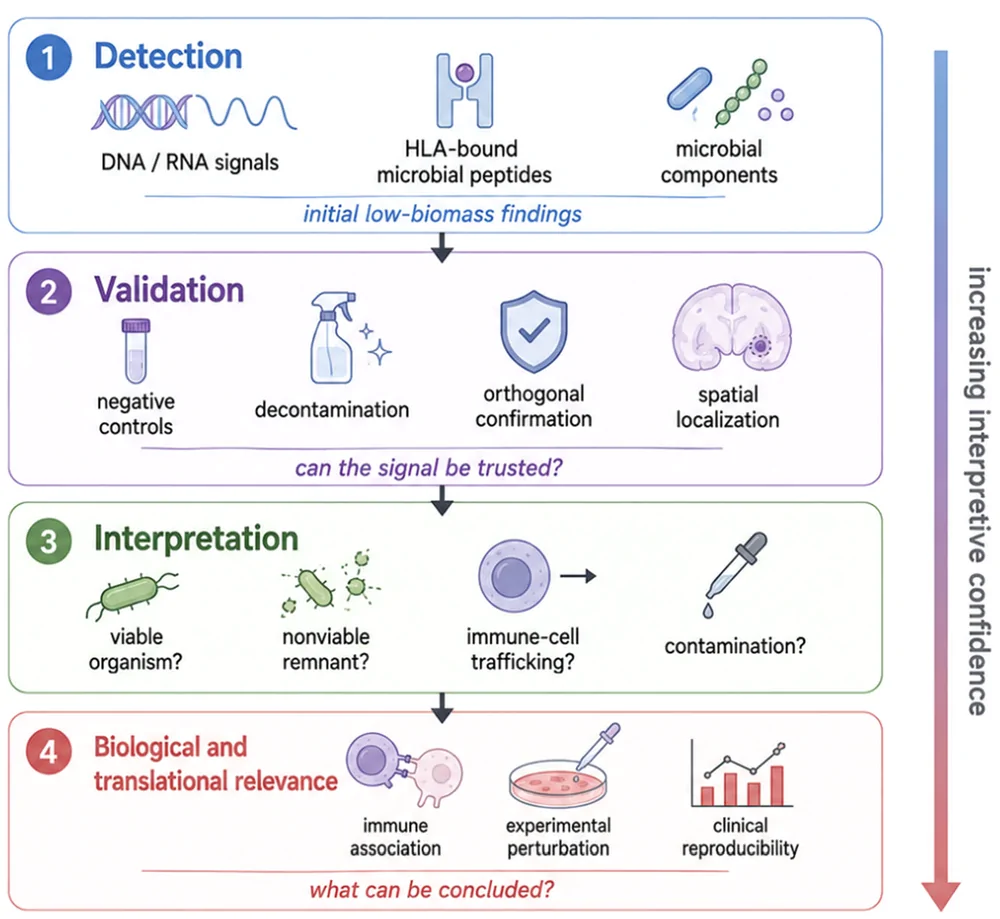
Framework for interpreting tumor‐associated microbial signals in glioblastoma. Interpretation proceeds from initial detection of microbial DNA/RNA, HLA‐bound microbial peptides, or microbial components to rigorous validation using negative controls, decontamination procedures, orthogonal confirmation, and spatial localization. Subsequent assessment should distinguish viable organisms from nonviable remnants, immune‐cell trafficking, or contamination. Biological and translational relevance requires functional association, experimental perturbation, and reproducibility across clinical cohorts.

## 9 Discussion and Clinical Implications: From Association to Translational Readiness

### 9.1 What Can be Inferred From Other Cancer Microbiome Models?

Microbial carcinogenesis is well established only for selected organism‐cancer relationships. In extracranial tumors, bacteria can contribute through chronic inflammation, genotoxicity, immune remodeling, or altered treatment response; these mechanisms provide a framework for GBM experiments but do not establish a bacteria‐driven origin for human glioblastoma (Ramadan et al. 2026; Garrett 2015; Riquelme et al. 2019).

### 9.2 Biomarker Readiness

No gut‐ or tumor‐derived microbial biomarker has been independently validated for GBM diagnosis, prognosis, treatment selection, or longitudinal monitoring. Small cohorts have reported taxonomic and diversity differences, but these results remain vulnerable to geography, diet, medication exposure, treatment, and tumor heterogeneity (Jiang et al. 2024, 2022; Green et al. 2024). Tumor‐associated microbial signals face an additional analytical barrier: a clinically useful assay must distinguish biological signal from reagent contamination and define whether the target is spatially localized, viable or nonviable, functionally relevant, and reproducible across centers (Morad et al. 2025; Eisenhofer et al. 2019; Fierer et al. 2025).

### 9.3 Therapeutic Readiness

Probiotics, defined microbial communities, dietary interventions, FMT, and microbial metabolites have modified immune or tumor‐related outcomes in preclinical glioma models, providing a rationale for mechanistic combination studies rather than evidence of clinical efficacy (Zhou et al. 2024; Wang et al. 2025; Li et al. 2025b). Clinical proof of principle for FMT‐mediated modulation of checkpoint‐inhibitor responses comes from melanoma and other extracranial cancers, not from GBM. These studies remain early and cannot be directly extrapolated to the profoundly immunosuppressive glioblastoma setting (Baruch et al. 2021; Duttagupta et al. 2026; Routy et al. 2023).

Microbial‐peptide vaccination and cross‐reactive T‐cell strategies remain similarly early. HLA‐bound bacteria‐associated peptides demonstrate biological feasibility, but candidate receptors or vaccines require proof of natural presentation, clinically meaningful tumor recognition, and rigorous off‐target testing (Naghavian et al. 2023; Bentzen and Hadrup 2019; Tagliamonte and Buonaguro 2022).

### 9.4 Current Clinical Studies, Safety, Standardization, and Regulation

Current prospective GBM microbiome studies are primarily observational, including THERABIOME‐GBM (NCT05326334), NCT03631823, and NCT06117930. They can define longitudinal associations and feasibility but do not constitute randomized evidence for a microbiome‐directed treatment (ClinicalTrials. gov 2026a, 2026b, 2026c). Safety is particularly important in GBM because corticosteroids, temozolomide, radiotherapy, surgery, and disease‐related immune dysfunction may increase infectious risk. FMT has transmitted multidrug‐resistant organisms to immunocompromised recipients, including a fatal infection, supporting rigorous donor screening, resistance testing, traceability, and pharmacovigilance in oncology research (DeFilipp et al. 2019; US Food and Drug Administration et al. 2019). For live biotherapeutic products or defined consortia, standardization should include strain identity, viability, dose, stability, potency, resistance determinants, virulence factors, and batch consistency. In the United States, FDA enforcement discretion for conventional FMT is limited to specified recurrent *Clostridioides difficile* settings and does not constitute routine authorization for oncology use (US Food and Drug Administration et al. 2022).

### 9.5 Patient Stratification and Trial Design

Future GBM trials should stratify or adjust for molecular tumor class, MGMT promoter methylation, disease stage, corticosteroid exposure, recent antibiotics, proton‐pump inhibitors, antiseizure medications, diet, body mass index, bowel function, and baseline immune status. Sampling should begin before major treatment exposures whenever feasible and continue longitudinally through chemoradiotherapy and recurrence. Initial interventional studies should prioritize safety, engraftment or exposure, and biological target engagement before efficacy endpoints are interpreted. The current evidence, translational readiness, principal barriers, and priority next steps for major microbiome‐related strategies in GBM are summarized in Table 2.

**Table 2:** Translational readiness of microbiome‐related strategies in GBM.

Strategy	Current GBM evidence	Translational readiness	Main barrier	Priority next study	Reference(s)
Gut microbial biomarker	Small observational cohorts	Low	No external validation; substantial confounding	Prospective multicenter validation in molecularly defined GBM	(Jiang et al. (2024); Jiang et al. (2022); Green et al. (2024))
Tumor microbial signature	Low‐biomass tissue signals	Low	Contamination and uncertain viability	Orthogonal spatial and viability confirmation	(Morad et al. (2025); Eisenhofer et al. (2019); Fierer et al. (2025))
SCFA/metabolite intervention	In vitro and preclinical models	Low	Source and physiologic exposure uncertain	Dose/exposure studies with tumor target engagement	(Zhou et al. (2024); Takenaka et al. (2019); Kay et al. (2025))
Probiotics/defined consortia	Mouse glioma models	Low	Strain specificity and product standardization	Reproducible defined‐product studies	(Keane et al. (2025); Wang et al. (2022); Li et al. (2025b))
FMT	No therapeutic GBM trial	Very low	Safety, donor variability, and regulation	Mechanistic and safety studies before GBM intervention	(Wang et al. (2025); Baruch et al. (2021); Duttagupta et al. (2026))
Microbial‐peptide immunotherapy	HLA peptide profiling and ex vivo evidence	Very low	Antigen source and off‐target reactivity	Patient‐specific HLA/TCR validation	(Naghavian et al. (2023); Kalaora et al. (2021); Bentzen and Hadrup (2019))
Bacteriophage modulation	Extracranial proof of concept	Conceptual	No validated bacterial driver in GBM; uncertain CNS delivery	Target validation and biodistribution in glioma models	(Ramadan et al. (2025b); Zheng et al. (2019))

## 10 Future Research Directions

### 10.1 Circadian, Glymphatic, and Melatonergic Integration of the Microbiota–Neuroinflammation Axis

An emerging research direction is the intersection between microbiota‐associated inflammation and the circadian–glymphatic–melatonergic network. Glymphatic dysfunction has been implicated in GBM‐related fluid and immune dynamics, while experimental sleep‐restriction data indicate that melatonin can preserve glymphatic function through aquaporin‐4 polarization at astrocytic end‐feet (Fiallo Arroyo and Leon‐Rojas 2026; Sun et al. 2025). These observations are not yet evidence that melatonin restores glymphatic clearance in human GBM, but they identify a tractable interface between sleep biology, astrocyte function, and the tumor environment. Age‐related decline in pineal melatonin may intersect with gut‐barrier integrity, systemic exposure to microbial products, and mitochondrial regulation, although these links remain indirect in GBM (Karasek 2004; Anderson and Maes 2020). Recent work proposes that nocturnal melatonin contributes to systemic mitochondrial resetting by favoring fusion over fission, consistent with broader models in which sleep provides a temporal window for redox and bioenergetic restoration. Whether this process measurably alters the GBM immune microenvironment remains unknown (Anderson 2026; Richardson and Mailloux 2023).

The local tryptophan–melatonin pathway within GBM and its microenvironment also warrants direct study. A proposed model links AhR activity to cytochrome P450‐dependent melatonin metabolism and an increased N‐acetylserotonin (NAS)/melatonin ratio. This proposal requires caution: CYP1A2 is best established as a melatonin hydroxylase, and conversion toward NAS is not the dominant canonical pathway (Mokkawes and de Visser 2023). NAS can activate TrkB signaling in neuronal models (Jang et al. 2010), while TrkB‐related signaling has been proposed to support glioblastoma stem‐like cell survival in broader mechanistic models (Anderson and Reiter 2019). The integrated AhR–CYP–NAS–TrkB pathway should therefore be treated as a hypothesis to test in GBM cells, stem‐like populations, astrocytes, microglia, and macrophages rather than as an established human‐tumor mechanism.

### 10.2 Bacteriophage‐Based Modulation of Tumor‐Associated Microbial Communities

Bacteriophages provide a conceptually different route to microbiome manipulation because they can target defined bacterial strains rather than broadly altering the intestinal ecosystem. Lytic phages can reduce selected bacteria, phage‐associated enzymes can disrupt biofilms, and engineered phage systems can serve as delivery or antigen‐display platforms. In colorectal cancer models, phage‐guided targeting of *Fusobacterium nucleatum* has augmented chemotherapy responses, providing proof of principle that a tumor‐associated bacterium can be manipulated therapeutically (Ramadan et al. 2025b; Zheng et al. 2019). Translation of bacteriophage strategies to GBM remains hypothetical. No viable bacterial driver has been reproducibly validated in human GBM, and CNS or blood‐tumor‐barrier delivery of therapeutic phages has not been established. Future studies should first validate a causal microbial target and then evaluate phage biodistribution, resistance, immune effects, and safety in immunocompetent glioma models.

## 11 Conclusion

Glioblastoma remains a uniquely difficult malignancy because diffuse invasion, molecular plasticity, myeloid‐dominant immune suppression, and systemic immune dysfunction limit the effectiveness of current therapies. The microbiota adds a plausible layer of host regulation, but the available evidence is uneven: mechanistic plausibility is broad, causal evidence is strongest in preclinical models, and human clinical evidence remains largely associative. The key distinction is therefore between a microbiota‐associated signal and a clinically established microbiota–GBM mechanism. Bacterial nucleic acids or peptides do not by themselves establish stable colonization; circulating metabolites do not establish a gut source; and a treatment effect after antibiotics, diet, or FMT does not automatically identify the responsible microbial pathway. Progress will depend on source attribution, orthogonal validation, perturbation‐and‐rescue experiments, longitudinal human sampling, and replication across centers. Microbiome‐based biomarkers and therapies are not currently validated for GBM. A clinically useful field will require evidence that a defined microbial feature is reproducible, mechanistically linked to a tumor or immune phenotype, adds information beyond established GBM variables, and can be modified safely. Framing future work around these evidentiary thresholds may allow microbiota research to contribute meaningfully to precision neuro‐oncology without overstating the current level of clinical readiness.

## Author Contributions

**Tareq Nayef AlRamadneh:** writing – review and editing. **Renuka Jyothi‐S:** writing – review and editing. **Priya Priyadarshini‐Nayak:** writing – review and editing. **Anima Nanda:** writing – review and editing. **Shaker Al‐Hasnaawei:** writing – review and editing. **Akanksha Bhatt:** writing – review and editing. **Ashish Singh‐Chauhan:** writing – review and editing. **Siya Singla:** writing – review and editing. **Manoj Kumar Mishra:** writing – review and editing.

## Funding

The authors have nothing to report.

## Ethics Statement

The authors have nothing to report.

## Conflicts of Interest

The authors declare no conflicts of interest.

## Data Availability

Data sharing is not applicable to this article as no datasets were generated or analyzed during the current study.
